# Sex hormones and immune system: Menopausal hormone therapy in the context of COVID-19 pandemic

**DOI:** 10.3389/fimmu.2022.928171

**Published:** 2022-08-02

**Authors:** Marina Averyanova, Polina Vishnyakova, Svetlana Yureneva, Oksana Yakushevskaya, Timur Fatkhudinov, Andrey Elchaninov, Gennady Sukhikh

**Affiliations:** ^1^ National Medical Research Center for Obstetrics, Gynecology and Perinatology named after Academician V. I. Kulakov of Ministry of Healthcare of Russian Federation, Moscow, Russia; ^2^ Peoples’ Friendship University of Russia, Medical Institute, Moscow, Russia; ^3^ A. P. Avtsyn Research Institute of Human Morphology, Laboratory of Growth and Development, Moscow, Russia

**Keywords:** sex hormones, steroids, immune system, menopausal hormone therapy, cytokines

## Abstract

The fatal outcomes of COVID-19 are related to the high reactivity of the innate wing of immunity. Estrogens could exert anti-inflammatory effects during SARS-CoV-2 infection at different stages: from increasing the antiviral resistance of individual cells to counteracting the pro-inflammatory cytokine production. A complex relationship between sex hormones and immune system implies that menopausal hormone therapy (MHT) has pleiotropic effects on immunity in peri- and postmenopausal patients. The definite immunological benefits of perimenopausal MHT confirm the important role of estrogens in regulation of immune functionalities. In this review, we attempt to explore how sex hormones and MHT affect immunological parameters of the organism at different level (*in vitro*, *in vivo*) and what mechanisms are involved in their protective response to the new coronavirus infection. The correlation of sex steroid levels with severity and lethality of the disease indicates the potential of using hormone therapy to modulate the immune response and increase the resilience to adverse outcomes. The overall success of MHT is based on decades of experience in clinical trials. According to the current standards, MHT should not be discontinued in COVID-19 with the exception of critical cases.

## Introduction

The new coronavirus infection (COVID-19/SARS-Cov-2) has quickly reached pandemic proportions following the initial outbreak in Wuhan (1, 2). A wide range of measures are being taken around the world with occasional declarations of emergency and restrictions on daily life. Over 2020–2021, the pandemic with over 160 million recorded cases of coronavirus disease (COVID-19) caused almost 3.5 million deaths (3). The situation continues to pose a threat to the world economy and human well-being. Efficient therapies for SARS-Cov-2 acute coronavirus infection represent an urgent global challenge to laboratory and clinical research.

Severe complications and high mortality of COVID-19 result from the massive cytokine storm that triggers inflammatory infiltration of the lungs and ends in the acute respiratory distress syndrome. The cytokine storm may also promote acute cardiac failure, secondary infections, generalized sepsis, and multiple organ failure, all of them potentially lethal. Prevention of the cytokine storm in SARS-Cov-2-infected individuals is pivotal. Steroid hormones, e.g. estrogens, are renowned anti-inflammatory agents. Cytokines, hormones, and neurotransmitters coordinate and integrate the immune, nervous, and endocrine functionalities through interactions with specific receptors in target cells (4). The majority of immune cells are known to express estrogen receptors (5). An association between estradiol intake (as a component of menopausal hormone therapy, MHT, as well as a component of hormone replacement therapy, HRT) and reduced risks of lethal outcome in SARS-Cov-2 has been demonstrated in a number of studies (6–8). At the same time, immune status of women receiving MHT has not been studied in detail. In this review, we attempt to explore how MHT affects immunological parameters of the body and what mechanisms are involved in its protective response to the new coronavirus infection. The review is based on PubMed searches (https://pubmed.ncbi.nlm.nih.gov/) using the keywords: COVID-19, SARS-CoV-2, coronavirus, novel coronavirus infection, autoimmunity, sex hormones, estradiol, progesterone, testosterone, immune response, menopausal hormone therapy, hormone replacement therapy, oophorectomy, postmenopausal women, immune aging.

### Sex-based differences in COVID-19 outcomes

The differences in hormonal status that distinguish men and women throughout their lives contribute to considerable immunological dimorphism reflected by skewed epidemiological profiles. The situation can be illustrated by reciprocal incidence proportions of autoimmune disorders and cancers: the former are more frequently diagnosed in women, and *vice versa* (9–11).

Female sex hormones, including estrogens and progesterone, act as regulators of innate and adaptive immunity; hence the higher flexibility of humoral and cell-mediated immune responses in women. A number of studies have come to conclusion that women show increased resistance to viral diseases and SARS-CoV-2 infections in particular (12).

Indeed, the incidence of severe cases and deaths of COVID-19 in women is lower (13, 14). In the International Severe Acute Respiratory and emerging Infections Consortium (ISARIC) World Health Organization (WHO) Clinical Characterization Protocol UK prospective observational study (CCP-UK) enrolling approximately 20,000 hospital patients with COVID-19 in early 2020, female lethality was lower by 20% (15).

Two previous outbreaks of zoonotic β-coronavirus encountered in this century showed similar epidemiological patterns. Among a total of 1,755 patients hospitalized during the 2002 outbreak of SARS-CoV in the Guangdong province of China, mortality rates constituted 13% for women and 22% for men (16). During the 2012 coronavirus epidemic in Saudi Arabia, the mortality rates were 23% and 52%, respectively (17).

As shown by the analysis of COVID-19 data from Italy, Spain, Germany, Switzerland, Belgium, and Norway, mortality rates in males exceed those in females for all age groups except under-20-year-olds (18). The established fact that adult men of all ages and women over 50 have the highest risks of severe complicated COVID-19 rekindles the point on the role of sex steroids in the clinical course of COVID-19 (19). Female immune system appears to produce a better coordinated and more flexible antiviral response with the overall impact on the morbidity, severity, and associated mortality. The trend may be explained by the modulatory effects of estrogens on leukocyte functionalities, both in circulating pools and resident populations of cells recruited from the bloodstream to peripheral tissues (12, 20–23). However, a number of reports emphasize the lack of difference in estradiol levels between deceased and surviving patients with COVID-19 (24, 25). Here we focus on the relationship between sex hormone levels and immune status of the body with a special regard to SARS-CoV-2 infection.

## Sex hormones as *chefs d’orchestre* for the immune system

### Estrogens define immunological parameters

Steroid hormones play important roles by tuning immune responses through modulatory effects on diverse cell populations representing both innate (neutrophils, macrophages/monocytes, natural killers, and dendritic cells) and acquired immunity (T and B cells) (4), including in immune-mediated diseases (26).

Sex hormones, which include estrogens, progesterone, and androgens, are produced in all humans; however their plasma levels, physiological duties, and target organs in men and women are different (27). In reproductive age women, estrogens and progesterone are produced cyclically by the ovaries, while small amounts of testosterone are produced by ovaries and adrenal glands. Estrogens are also produced locally by aromatization of androgens in adipose tissue, bones, and mammary glands. In men, estrogen blood levels are maintained through aromatization of testosterone in peripheral tissues, whereas Leydig and Sertoli cells of the testes are engaged in local synthesis (27).

Endogenous estrogens include estrone (E1), 17β-estradiol (E2), and estriol (E3) (28). Estradiol is the predominant and most biologically active estrogen. Estradiol is a physiological derivative of testosterone, whereas estrone is derived from androstenedione; the syntheses are catalyzed by aromatase. Estrone, which prevails in postmenopause, has weaker effects compared with estradiol.

Estrogen receptors are expressed in all immune cells and participate in transcriptional regulation (12, 20–23). Estrogen receptors fall into two types: intracellular (ERα and ERβ) and membrane-bound (G-protein coupled estrogen receptor, GPER). Accordingly, the routes of estrogen signaling involve genomic and non-genomic options. Genomic estrogen signaling involves interactions of genomic DNA with ligand-bound ER, either direct (classical) or mediated by other transcription factors, whereas non-genomic estrogen signaling acts by triggering cytoplasmic protein phosphorylation cascades (29). The classical genomic estrogen signaling (characteristic of steroid hormones in general) consists of the following steps (1): the hormone enters the cytoplasm and meets its nuclear receptor (2); the hormone-receptor complex is translocated to the nucleus (3); the complex binds specific recognition elements in promoter regions of effector genes (30). However, some effects of estrogens are too fast to be a consequence of gene expression (which takes time invariably). Instead, the fast effects of estrogens result from non-genomic signaling routes triggered by engagement of the membrane-bound GPERs (31–34). Such effects include the estrogen-mediated activatory phosphorylation of the endothelial nitric oxide synthase (eNOS) (35); similar non-genomic effects have been reported for other steroid hormones. Thus, immune cells have multiple routes of responding to the circulating estrogen levels, and exploit them in accordance with receptor profiles expressed by particular cells (36, 37).

The net effect of estrogens on immune functionalities is anti-inflammatory. Studies show that physiological estrogen levels in premenopausal women suppress the release of pro-inflammatory cytokines, notably interleukins IL-6 and IL-8, and tumor necrosis factor α (TNF-α) (38). By contrast, low physiological estrogen levels in postmenopausal women fail to counteract the release of pro-inflammatory cytokines. Quite indicatively, the elevated levels of IL-1, IL-6, and TNF-α, encountered in postmenopause, can be effectively mitigated by MHT (39, 40).

Cellular mechanisms of the anti-inflammatory action of estrogens are likely to involve diverse leukocyte populations of the body. Estrogens have been shown to regulate cell numbers and functional activities of neutrophils by affecting the release of many chemokines (e.g. monocyte chemoattractant protein MCP-1) and cytokines (TNF-α, IL-1β, and IL-6) (41). The suppressive effect of estrogens on the production of TNF-α, IL-1β, and IL-6 by neutrophils and macrophages has been independently confirmed in rats (42), mice (43), and humans (44).

The protective effect of estrogens on polymorphonuclear leukocytes involves activation of the regulatory pathway controlled by the prominent anti-inflammatory protein annexin A1. The response to female steroids in neutrophils is accompanied by a rapid increase in annexin A1 levels. Depletion of this protein by immunoneutralization or genetic modification abolishes the mitigating effect of estrogen on neutrophil extravasation both *in vitro* and *in vivo* (45) (**Table 1**).

**Table 1:** Immunity-related effects of sex hormones observed mainly *in vitro*.

Methods/Conditions	Applying	Concentration/Manipulation	Effect	Ref.
***Estrogens***				
Polymorphonuclear cells or whole blood aliquots incubated with E2	*in vitro*	17 β-E2 (5 ng/ml)	AnxA1 mobilization. The appearance of the phenotype: AnxA1^hi^CD62L^lo^CD11b ^lo^,	(45)
LPS-induced inflammation (10 ng/ml) on mouse embryonic fibroblast cells	*in vitro*	17 α-E2 or 17 β-E2 (10 µM)	↓TNF-α, ↓IL- *6* ↑IL-*4*, ↑IL*-6ra* 17 α-E2 and 17 β-E2 down-regulate NFκB-p65 expression	(46)
Mouse and human peripheral blood monocytes/macrophages activated by S. aureus	*in vitro*	Pretreatment with 17 β-E2 (10^-7^ М)	17 β-E2 inhibits the NF-κB pathway upon activation of S. aureus monocytes, also ↓TNF-α, ↓IL-1β, ↓IL-6 and ↓GM-CSF, ↓TLR2, ↑IL-10, ↑IL-27.	(47)
Whether E2 inhibits NFκB signaling in rat carotid injury models and in TNF-α treated rat aortic smooth muscle cells	*in vitro*	17 β-E2 (10 ^-7^ M)	E2: ↓inflammation in rat aortic smooth muscle cells by promoting synthesis of IκBα, a direct inhibitor of NFκB activation, and by directly inhibiting NFκB binding to inflammatory gene promoters.	(48)
Monocytes and neutrophils from blood of premenopausal women who had not previously used hormone therapy. Monocytes were pre-incubated with 17 β-estradiol for 24 hours and then treated or untreated with LPS for 12 hours (LPS (10 ng/ml))	*in vitro*	17 β-E2 (10 ^-8^ M)	E2: attenuates LPS-induced expression of CXCL8 in monocytes. Treatment of monocytes with E2 prior to LPS administration ↓CXCL8 signaling and protein production. The ability of LPS-activated monocytes pretreated with E2 to mobilize neutrophils was impaired.	(49)
Monocyte-derived macrophages were obtained from healthy premenopausal women and treated with E2. For LPS stimulation experiments cells were pre-treated with E2 for 24 hours followed by LPS administration for 12 hours (LPS (10 ng/ml)).	*in vitro*	17 β-E2 (100 nM)	Activation of macrophages by LPS: ↓κB-Ras2 expression. Pretreatment of human macrophages with E2: ↓LPS-induced TNF-α expression due to ↓activation of NF-κB. E2: suppressed NF-κB activation *via* κB-Ras2 induction.	(44)
Effect of 17 β-estradiol on gene expression in human lung epithelial cell line A549	*in vitro*	17 β-E2 (from 37 nM to 144 nM), exposure period 24 hours	E2: ↓ levels of cellular ACE2 mRNA and TMPRSS2 mRNA.	(50)
THP-1 cells were infected with tachyzoites of T. gondii strain RH. Stimulation was performed with E2	*in vitro*	17 β-E2 (40 nM)	T. gondii: ↑ERα, ↑ERβ, ↓ prolactin receptor (PRLR); E2: ↓PRLR;	(51)
Macrophages derived from human peripheral blood monocytes activated by LPS (100 ng/ml, M1) or IL-4 (15 ng/ml, M2)	*in vitro*	17 β-E2 (10^-11^ M); and xenoestrogens: bisphenol A (BPA) 10^-6^ M, DEHP (di-ethyl-2-hexyle phthalate) 10^-6^ M and DBP (di-n-butyl phthalate) 10^-6^ M in combination with selective antagonists ERα or ERβ.	E2 stimulated the migration of M2 macrophages. Xenoestrogens: M1: ↑IL-10 and ↓IL6. M2: ↓IL10, ↓IL6, ↓TNF-α and ↓IL1β	(52)
**Progesterone**				
Evaluation of the effect of progesterone on DC in rats after LPS stimulation (5 µg/ml)in the ranges covering physiological and pharmacological concentrations.	*in vitro*	Progesterone	Progesterone treatment of LPS-activated mature bone marrow DC: ↓TNF-α and ↓IL-1β production in a dose-dependent manner, but did not affect IL-10. Progesterone treatment: ↓CD80 and the ↓MHC class II RT1B molecule, ↓ DC-stimulated T-cell proliferation, ↓ability of mature rat bone marrow cells to drive pro-inflammatory responses.	(53)
THP-1 cells were infected with tachyzoites of T. gondii strain RH. Stimulation was performed and progesterone	*in vitro*	Progesterone (40 nM)	T. gondii: ↑ERα, ↑ERβ, ↓ prolactin receptor (PRLR); Progesterone: ↓PRLR, ↓ERβ expression and ↑ERα expression;	(51)
**Other**				
THP-1 cells were infected with tachyzoites of T. gondii strain RH. Stimulation was performed with prolactin	*in vitro*	Prolactin (200 ng/ml)	T. gondii: ↑ERα, ↑ERβ, ↓prolactin receptor (PRLR); Prolactin: ↓ERα and ↓ERβ.	(51)
Macrophages of the spleen and peritoneal macrophages at baseline and after LPS stimulation	*in vitro*	Ovariectomy	Splenic macrophages: ↓IL-1β, ↑IL-10 Peritoneal macrophages: ↓TNF-α, ↓IL-1β, ↓IL-10), ↑TGF-β Ovariectomy reduced urea production in both subpopulations of LPS-stimulated macrophages. After LPS stimulation compared with sham-operated animals: Splenic macrophages: ↓TNF-α, ↑IL-10 Peritoneal macrophages: ↓IL-1β, ↓TGF-β	(54)
♀ and ♂ mice (8–9 weeks, 5 and 8–10 months, 18–20 months) were intranasally infected with various doses of SARS-CoV	*in vitro* *in vivo*	Serum estradiol concentration was measured by ELISA	♂ mice were more susceptible to SARS-CoV infection compared to ♀ of the same age. The degree of gender bias towards SARS-CoV infection increased with age. Increased susceptibility of ♂mice to SARS-CoV has been associated with elevated virus titers, increased accumulation of inflammatory macrophages and neutrophils in the lungs. Sex differences did not depend on the response of T and B cells. Ovariectomy or treatment of ♀ mice with an estrogen receptor antagonist increased mortality.	(55)
Peritoneal macrophages obtained from young (2 months) and aging intact middle-aged rats (16 months): male and female	*in vitro* *in vivo*	N/A	♀ middle age compared to young: ↓ IL-1β, ↓IL-6, ↓ERα, ↓ systemic level E2 ♂ middle age compared to young: ↑IL-1β, ↑IL-6 ♀ of middle age compared to ♂: ↑IL-6, ↑IL-1β, ↑TNF-α and ↑NO	(56)

↑ and ↓ - up- and down-regulation, respectively.

E2 –estradiol.

LPS, lipopolysaccharide N/A, not available.

Monocyte-macrophage lineages are also highly responsive to estrogens. Many studies identify monocyte populations as the key anti-inflammatory effector of estrogens (57, 58). Estrogens inhibit expression of chemokine receptors CCR2 and CXCR3 in monocytes, thereby reducing their sensitivity to pro-inflammatory factors (59–61) (**Table 2**). Elevated blood levels of 17β-estradiol promote expression of anti-inflammatory markers in monocytes (66) while inhibiting the production of pro-inflammatory cytokines by these cells (67–69). More specifically, estrogens can modulate macrophage phenotypes (polarization status) in favor of anti-inflammatory profiles (70, 71). Toniolo et al. have demonstrated decreased estrogen levels observed in postmenopause negatively affects the macrophage capability of polarization towards anti-inflammatory phenotypes in response to microenvironmental stimuli (72).

**Table 2:** Immunity-related effects of sex hormones observed mainly *in vivo*.

Methods/Conditions	Applying	Concentration/Manipulation			Effect		Ref.
***In vivo***						
Mouse blood monocytes treated in culture and *in vivo*	*in vitro* *in vivo*		MCP-1/JE and MIP-1a; 17 β-E2 (2.5 µg); tamoxifen (4-hydroxy-tamoxifen) (2.5 μg).		Estrogens and tamoxifen: ↓CCR2, ↓CXCR3 in monocytes. Estrogens reduced monocyte chemotaxis in the presence of MCP-1/JE. Estrogens suppressed the ability of monocytes to respond to certain chemokines	(59)
Acute lung injury was induced by intratracheal instillation of bacterial LPS in male, female, and ovariectomized mice.	*in vitro* *in vivo*		17 β-E2 (50 mg/kg in 400 μl PBS) was administered intraperitoneally 1 hour before LPS administration		E2: ↓IL-6 and ↓IL-1β both *in vivo* and *in vitro*.	(62)
Effect of viral influenza A on the immune system of mice. 3 groups of female mice: with intact gonads, with gonadectomy and with gonadectomy on the background of hormone replacement therapy (HRT)	*in vivo*		Hormone capsules were left empty (placebo) or contained testosterone, dihydrotestosterone (DHT), or 17β-E2.		E2 influenced the kinetics of viral replication, but ↓TNF-α and ↓CCL2 in the lungs of mice with intact gonads and mice with gonadectomy. Mice with gonadectomy and low circulating E2 levels tolerate infection worse and have higher pro-inflammatory responses than females with intact gonads or on HRT.	(63)
Study of the effect of estrogens on the number and cytotoxic activity of 6 weeks female mice NK cells	*in vivo*		17 β-E2 (100 µg/kg/day)		E2: ↑ the number of NK cells, but ↓ their cytotoxicity	(64)
Evaluation of the effect of selective estrogen receptor modulators raloxifene (ral), lasofoxifene (las) and bazedoxifene (bza) on T-lymphopoiesis and inflammation. The study was conducted on mice after gonadectomy.	*in vivo*		Subcutaneous injections of 17β-estradiol-3-benzoate (E2; 1 µg/mouse/day, ral (60 µg/mouse/day), las (4 µg/mouse/day, or bza (24 µg/mouse/day.		Treatment with las or bza does not affect T-lymphopoiesis or T-dependent inflammation. E2: ↓ thymus mass and ↓ proportion of early progenitor T cells, while ↑ population of more mature T cells in the thymus. E2 also ↓T-cell dependent delayed-type hypersensitivity reaction.	(65)

↑ and ↓ - up- and down-regulation, respectively.

E2 –estradiol.

Administration of exogenous estradiol (E2) to male or ovariectomized female mice significantly reduced the expression of pro-inflammatory cytokine IL-1β by peritoneal macrophages *in vivo* (73). Zhang et al. reported alleviation of inflammatory response in macrophage cell line RAW264.7 by estradiol; the mechanism involved a reduction in expression and secretion of IL-1β (74). In a study by Stanojević et al., stimulation with endotoxin (lipopolysaccharide, LPS) reduced secretion of IL-1β and TNF-α by peritoneal macrophages in ovariectomized mice (54). However, no decrease in IL-1β production after treatment with estradiol was observed in monocytic macrophages derived from women in postmenopause (52). Ćuruvija et al. showed that LPS-stimulated peritoneal macrophages of middle-aged female rats with significantly reduced levels of circulating estradiol secrete less IL-1β than matched cells of young females (56). Galván-Ramirez et al. demonstrated that immortalized monocytic cells THP-1 treated with estradiol prior to stimulation by exposure to *Toxoplasma gondii* have decreased rates of production of the pro-inflammatory cytokine IL-12 (51). Estrogens have been also shown to suppress production of major neutrophil chemoattractants CXCL1, CXCL2, and CXCL3 within inflammatory foci in rodent models of colon (75), lung and vascular injury (48). Estradiol has been also demonstrated to inhibit the release of a potent neutrophil chemoattractant, chemokine CXCL8, by human monocytes *ex vivo* (49). Myeloid lineages isolated from ERα knockout mice (*Esr1* ^-/-^) showed poor performance in macrophage clearance tests and defective polarization of macrophages from classically activated (pro-inflammatory) to alternatively activated (anti-inflammatory) phenotypes (76).

A number of studies explore molecular mechanisms of the estrogen effects on cytokine production. The decrease in cytokine production under the action of estrogens apparently involves inhibition of NF-κB signaling pathway. Pre-treatment with estrogens has been shown to interfere with NF-κB signaling and ultimately inhibit the LPS-induced production of TNF-α by human macrophages *in vitro* (72). Santos et al. demonstrated that estrogens mitigate the LPS-induced inflammation through inhibition of the NF-κB–p65 axis in embryonic fibroblasts (46, 59, 77). Besides, estrogens are capable suppressors of the non-receptor Bruton’s tyrosine kinase (BTK) essential for monocyte functionalities and the interleukin-1 receptor-associated kinase IRAK2 at transcriptional level (78). *In vitro* exposure to estrogens mitigates the functional performance of immune cells challenged with LPS/interferon-γ: the production of TNF-α and IL-10 decreases, reflecting the interference of estrogens with NF-κB signaling *via* both genomic and non-genomic mechanisms (44). In particular, estrogens boost the production (48) and prevent the degradation of IκB-α — the chief endogenous NF-κB inhibitor (44).

Estrogens are also capable of influencing lymphocytes, albeit in this case the effects are obviously multidirectional. The available experimental findings advocate both pro- and anti-inflammatory effects. For instance, estrogens have been confirmed to modulate negative selection of the high-affinity autoreactive B cells and meddle with their functionalities, orchestrating a Тh 2 type response (22). Estrogens have been also shown to suppress thymopoiesis (65), promote T cell activation (79), and stimulate NF-κB signaling which controls numerous genes of immune response, cell cycle, and apoptosis (80). Estrogens also promote the synthesis of IL-1, IL-10, and interferon-γ by lymphocytes (81, 82), while supporting Th1 and Th17 differentiation (83), regulatory Т (Treg) cell maintenance, and the expression of immunosuppressive gene *FoxP3* (84–87). According to other studies, estrogens reinforce the immunosuppressive functionalities of Treg cells (88, 89) and boost the expression of chemokine receptors CCR1-5 (90), as well as the levels of chemokines MCP1, MCP5, eotaxin, and SDF1β (81, 91). On the other hand, estrogens stimulate the production of anti-inflammatory cytokines (e.g. IL-4 and IL-10) by CD4+ T helper cells. Estrogens also reduce the production of IL-17 by pro-inflammatory helper cells Th17 and stimulate Treg cell proliferation thus facilitating immune tolerance (92). Exposure of normal killer cells (NK cells) to estradiol *in vitro* promotes secretion of interferon γ by these cells and reinforces their cytotoxicity (93). At the same time, estradiol has been shown to suppress the expression of surface activation markers and FAS ligand by NK cells while inhibiting secretion of granzyme B serine protease by these cells in murine model (64).

Apart from *in vitro* studies, the immunity-related effects of estrogens were tested in a number of pro-inflammatory disease models *in vivo*. In the majority of experimental models, the anti-inflammatory effects of estrogens predominantly involved innate immunity: the reduced production of pro-inflammatory cytokines (e.g. IL-6, IL-1β, and TNF-α) by monocytes and macrophages along with chemokine production inhibition alleviated monocytic infiltration of inflammatory foci.

In pre-clinical studies using influenza A virus infection models, estrogens exhibited potent immunomodulatory effects leading to a more balanced innate immune response in the lungs, associated with reduced local levels of pro-inflammatory cytokines and reduced chemokine reactions before the onset of clinical symptoms (94–96).

In a model of acute pneumonia induced by instillation of bacterial LPS, male and ovariectomized female mice showed increased infiltration of the lungs with polymorphonuclear cells producing high amounts of IL-6, IL-1β, and the inter-cellular adhesion molecule 1 (ICAM-1); these symptoms were reduced upon exogenous administration of estradiol (62).

A major influence of estrogens on immune response in humans involves their meddling with the neutrophil recruitment to acute inflammation foci. Several studies identify two basic routes of such effects (1): control of the neutrophil chemotaxis and (2) modulation of the interactions between neutrophils and endothelial cells (97).

Acute lung injury in ovariectomized mice was successfully treated with estrogen replacement therapy (62). Administration of estrogen receptor antagonists (or ovariectomy) increased the lethality of SARS-CoV infection among female mice (55). Estrogens have been also demonstrated to play an important role in protection of the lung tissue through spatial confinement of local inflammation. This capacity was confirmed by *in vivo* experiments with administration of LPS to male or ovariectomized female mice. Despite an initial boost in the levels of IL-1β in response to LPS, administration of estradiol reduced both the albumin levels and the degree of LPS-induced lung injury (16, 62).

### Progesterone impacts immune responses

Progesterone is another important immunomodulatory and anti-inflammatory hormone. Its cognate receptors are expressed by multiple cell types of the immune system, including macrophages, dendritic cells, lymphocytes, mast cells, and eosinophils. Progesterone also binds and activates glucocorticoid and mineralocorticoid receptors, which results in suppressed production of pro-inflammatory IL-1β and IL-12 cytokines by macrophages and dendritic cells (98). According to a number of studies, progesterone inhibits T cell proliferation, promotes apoptosis, facilitates production of IL-4 while reducing production of interferon β and IL-17, and also inhibits Th1 and Th17 activities while supporting Treg cell differentiation (99, 100).

Progesterone has a broad spectrum of anti-inflammatory effects. After exposure to progesterone, macrophages and dendritic cells have inferior activation status and produce less IL-1β and TNF compared with untreated cells (53, 101). Progesterone is also known to facilitate proliferation of Treg cells and thus support immune tolerance (92). Exposure to progesterone promotes expression of FIZZ1 and YM1 (the alternatively activated anti-inflammatory macrophage markers) and inhibits expression of the inducible nitric oxide synthase (iNOS) with a concomitant decrease in NO production in bone marrow-derived murine macrophages. The Toll-like receptor (TLR) and NF-κB signaling pathways can be antagonized by progesterone-mediated effects. Cytokine storm is the climax of severe COVID-19 and its mechanisms clearly have a TLR-dependent component. Accordingly, the role of TLR signaling pathways in COVID-19 pathogenesis represents a continuous research focus (102, 103). Despite the predominant implication of TLR7/8, other receptors of this family appear to be involved as well. For instance, the TLR3/TLR4 double-knockout mice are more susceptible to SARS-CoV-2 infections (104) and many studies emphasize the significance of TLR4 in the cytokine storm development (105).

Exposure of human NK cells to progesterone mitigates their activation and inhibits production of interferon γ by these cells through caspase-dependent apoptosis. Progesterone also modulates Th cell-mediated responses by promoting a Th1-to-Th2 shift in Th phenotypes and facilitating production of anti-inflammatory cytokines (e.g. IL-4 and IL-10) by Th cells. In addition, progesterone inhibits production of pro-inflammatory cytokines(e.g. IL-1β and IL-12) by dendritic cells (98, 106).

### Androgens impact immune responses

Testosterone exerts immunosuppressive action targeted at several constituents of the immune system, including effector cells of innate and adaptive immunities. Testosterone can inhibit production and release of pro-inflammatory cytokines (IL-1β, IL-6, TNF-α, interferon γ, and IL-12), while promoting production of anti-inflammatory cytokines (IL-10 and IL-4) (107). Androgens have been shown to suppress Th1 lineages and support Th2 differentiation, while inhibiting В lymphopoiesis and production of antibodies by B cells (108). A number of studies confirm the overall anti-inflammatory effect on androgens using experimental models of autoimmune and inflammatory diseases, progression of which can be slowed down by testosterone administration. Large prospective studies associated lower testosterone levels and increased estradiol-to-testosterone ratio in men with severe course of COVID-19 and high levels of pro-inflammatory cytokines (109). However, this association cannot be considered a direct evidence of the pro-inflammatory effect of estradiol in men, as it likely reflects a reduction in testosterone levels associated with visceral obesity — a risk factor on its own.

The main histocompatibility complex (MHC types I and II) engaged in the pathogenic antigen presentation plays a pivotal role in immune response. Testosterone is known to reduce the levels of MHC II expression on dendritic cells, while estrogens exert the opposite action by increasing the MHC II expression levels (110). The sex-based difference in immunity reactions may be related to Х-chromosome localization of certain immunoregulatory genes (9). At least, *FoxP3* and *CD40L* genes are expressed at higher levels in women. The variable patterns of X inactivation in immune cells and the pleiotropic functional spectrum of many genes provide a favorable playground for sex hormones to finely orchestrate the immune system capacity of breaking tolerance to exogenous or endogenous agents (9, 111).

Sex hormones not only control the reproductive system, but also largely tune the immunity. The hormones regulate immune response in the whole diversity of its aspects and forms (innate and adaptive, humoral and cell-mediated), so that any flaws in the mechanisms of such regulation contribute to the development of immune-mediated diseases, including autoimmune conditions (98, 112–117). Although the exact molecular mechanisms of the immunological impact of sex hormones are not yet fully understood, studies show that sex hormones profoundly control development, homeostasis, gene expression, and signaling in T and B lymphocytes, monocytes, macrophages, dendritic cells, and granulocytes, deeply affecting their functionalities under normal and pathological conditions.

### Non-immune effects of sex hormones

Among their diverse systemic effects, sex steroids are known to interfere with local immunityby stimulating local immunocompetent cells and modifying the properties of epithelial barrier. Although steroid receptors are expressed by many mammalian cell types, the action of sex steroids on mucous membranes of the genital tract is surely the main focus (118). Estradiol promotes secretion of leukocyte protease inhibitor and β-defensin-2 by human uterine epithelial cells thus enhancing their antimicrobial properties (119). With the onset of menopause, the barrier function of the endometrium declines, which is associated with changes in composition of subepithelial lymphocyte populations (118), thinning of the epithelium (120), and disruption of epithelial cell junctions, especially those involving cadherins (121). Vulnerability of other types of cell junctions in the uterine epithelium during postmenopause is questionable, although certain pathogens, such as HIV, have been reported to facilitate destruction of tight and adherens junctions (122). Noteworthy, estradiol inhibits secretion of IL-6, IL-8, and MIF by uterine epithelium in response to TLR3/4 stimulation (119), as well as the INFγ-induced gene expression, while progesterone has the opposite effect (123). The effect of sex steroids on other mucous membranes is less pronounced. Decreased 17β-estradiol and progesterone levels are accompanied by decreased salivary levels of IgA and higher incidence of upper respiratory tract infections (124). In addition, salivary levels of secretory IgA in women are known to be significantly higher than in men (125, 126); although the menstrual cycle-related dynamics are negligible. Estrogens promote IgA transport across the epithelia, thus contributing to the barrier function of mucosa, for instance, in the intestine (127).

The effects of sex steroids on mucous membranes of digestive tract are widespread. For instance, estradiol administration reduced the symptoms of eosinophilic esophagitis (128), a typical dysfunction of the epithelial barrier. At the same time, estrogens, in contrast to testosterone, can impede wound healing in the oral cavity (129).

A human herpesvirus 2 (HSV-2) vaccine administered intranasally against the background of E2 estradiol ensures more pronounced Th17 responses, longer persistence of CD4+ T cells, and higher numbers of memory Th-cells in the upper respiratory tract mucosa-associated lymphoid tissue (130). The diverse effects of sex hormones are determined by robust expression of their receptors in a variety of mammalian cell types.

### Estrogens attenuate the pro-inflammatory cytokine storm

The fatal outcomes of COVID-19 are due to the intrinsically high reactivity of the innate wing of immunity. Under conditions of severe respiratory illness, the innate immune system overreacts by critical hypercytokinemia and massive migration of the activated immune cells to the lungs. The patients die not of the minor tissue damage caused by viral replication *per se*, but as a consequence of generalized devastating immune response with characteristic off-scale burst in systemic levels of pro-inflammatory cytokines leading to the acute distress-syndrome and multiple organ failure (92). These devastating consequences can be considerably mitigated by estrogens at different planes of SARS-CoV-2 infection: from increasing the antiviral resistance of individual cells to counteracting the pro-inflammatory cytokine production.

Angiotensin-converting enzyme 2 (ACE2) was identified as a unique cognate receptor for SARS-CoV-2, key for penetration of the virus into human cells. Patterns of ACE2 expression in human body (in terms of distribution and intensity) play pivotal role in the course of the infection accomplished through the binding of ACE2 to the SARS-CoV-2 spike glycoprotein (S-protein) (131–133). ACE2 protein is a membrane-bound aminopeptidase expressed in a variety of organs and tissues including heart, intestine, kidneys, lungs, lymph nodes, and ovaries (134). ACE2 is predominantly expressed by endothelial cells, as well as myocardium, intestinal mucosa, and type II pneumocytes — the pulmonary surfactant-producing spherical cells found in lung alveoli (135). ACE2 is also expressed by other cell types and structures of the respiratory tract, from nasopharyngeal mucosa to the transient secretory cells of the bronchi. The difference of ACE2 protein expression patterns observed in men and women may partially explain the sex-based differences in COVID-19 morbidity and mortality (136–139). The ACE2 receptor protein is abundantly expressed in hormone-producing organs and structures, notably in testes, the thyroid, and adipose tissue, and to a lesser extent also in adrenal glands, while the tendency for its increased expression identified in males and older individuals is consistent with the higher morbidity observed for these groups (131).

Estrogens and particularly their anti-SARS-CoV-2 immunomodulatory effects are currently a close focus. The acute respiratory distress syndrome (ARDS) is prevalent in severe COVID-19. Pathogenetic routes of this condition vary; one of them involves dysfunction of alveolar epithelial cells leading to the gas exchange disruption. The favorable outcome in ARDS is thought to depend on the effectiveness of alveolar fluid clearance (AFC) (140), which involves active transport of sodium ions (141). Sex steroids likely participate in regulation of this process, as women with ARDS show higher AFC rates and get more favorable prognosis than men (140). The same sex-based difference has been observed in preterm infants with respiratory distress syndrome (142). Several experimental studies show that administration of estrogen and progesterone supports the synthesis of pore-forming α-subunit of ENaC and Na,K-ATPase thus stimulating sodium transport and ultimately AFC, which may provide important link in the treatment of ARDS (143–145). Another study argues that the apparent beneficial effect of estradiol in LPS-induced acute lung injury may involve the PI3K/Akt/SGK1 signaling pathway activation (146). In addition, administration of 17β-estradiol has been shown to prevent the development of age-related changes in the lungs in female mice: it mitigates cell death, inhibits MMP2 expression, and restores interalveolar septa (147).

Estrogens have been shown to inhibit expression of the transmembrane serine protease 2 (TMPRSS2) in various cell lines (50). TMPRSS2 is required for the activation of spike protein in some coronaviruses, notably in SARS-CoV and SARS-CoV-2 (148). In human respiratory airways SARS-CoV-2 virus infects cells through interaction of the capsid S-protein with ACE2 (149). The inhibition of TMPRSS2 expression by estrogens can prevent the infection since TMPRSS2 and ACE2 are co-expressed, i.e. “interact” at transcriptional level (150). A recent study by Baristaite et al. shows that estradiol may alleviate the symptoms by regulating *ACE2* and *TMPRSS2*, as their expression decreased upon 17β-estradiol treatment of A549 human lung epithelial cells *in vitro*.

The antiviral potential of estrogens is partially explained by its direct impact on transcription (mediated by nuclear receptors), as many genes known to participate in immune response and inflammation have estrogen-responsive elements in their promoter regions (151). Apart from that, estrogens counteract vasoconstriction by stimulating the nitric oxide synthesis and reducing the intracellular calcium levels in vascular smooth muscle cells (152). Estrogens are thought to mobilize the resting endothelial progenitors to proliferating pools, as well as to reduce the rates of apoptosis among endothelial cells (153). Besides, estrogens exert anti-inflammatory action on the endothelium by inhibiting leukocyte chemotaxis and formation of reactive oxygen species through activation of the renin-angiotensin-aldosterone system. Estrogen deficiency is accompanied by elevated levels of renin and increased expression of angiotensin receptor 1 with ensuing vasoconstriction and pro-inflammatory cytokine shift (154). These negative effects can be neutralized by exogenous estrogens at the non-genomic level.

Toll-like receptor 7 (TLR7) is expressed on dendritic cells (155). Berghöfer et al. demonstrated that, upon stimulation with TLR7 ligands, peripheral blood plasmacytoid dendritic cells of women produce more interferons type I than corresponding cells of men (156). The authors also observed the loss of TLR7-mediated responses in plasmacytoid dendritic cells during postmenopause and showed that it could be partially rescued with MHT. The TLR7 encoding gene is located on X chromosome (103), which suggests sex-based differences in the effectiveness of antivirals (111), including those against SARS-CoV-2, consistently with clinical observations (157). Female dendritic cells, monocytes and B lymphocytes tend to avoid inactivation of the *TLR7* second copy (158), leading to increased *TLR7* dosage and ultimately to higher levels of type I IFN (IFN-I) (158). The higher levels of IFN-I production by dendritic cells observed in women have been suggested to depend on estrogen levels (159–161). At the same time, dendritic cells of female mice transplanted into males continued to produce higher levels of IFN-I, which indicates a strong relationship of this phenomenon to the number of X chromosomes i.e. *TLR7* copy number (161). A prominent role of estrogens in regulation of TLR-mediated responses has been confirmed by several studies (162, 163).The involvement of TLR7 pathway in systemic response to SARS-CoV-2, genomic sequences of which can activate the endosomal TLR7/8, has been supported by multiple evidence (164). The concomitant activation of TLR7 RNA sensor pathways, followed by activation of NFκB signaling, promotes secretion of IFN-I, IFN-γ, and IFN-λ3 within 48 h of active SARS-CoV-2 infection (165). The TLR7/8 agonist imiquimod has been shown to stimulate the production of TNF-α, IL-1, IL-2, IL-6, IL-8, IL-12, as well as IFN-α (166).

The relationship between TLR7 levels and sex-based differences in the clinical course of SARS-CoV-2 infection has been also demonstrated in studies featuring male patients with deleterious variants of TLR7 (Xp22.2) (167) leading to compromised TLR7 activation and severe COVID-19 (168).

But even accounting for both the unfavorable TLR7 variants in men (169) and the stimulating effect of estrogens, the enhanced TLR7 expression in women is difficult to explain. The discovery of X-chromosome inactivation avoidance makes an important point, as about 15–20% of active genes on X chromosome have been shown to escape inactivation of the extra copy (170). The degree of avoidance, as well as the tissue-specific signatures of non-inactivated sequences, require further investigation (171, 172). X-linked differences in antiviral immunity have been described for SARS-CoV-2, hepatitis C virus, and HIV.

As long as estrogens suppress the production of pro-inflammatory cytokines, they can have a decisive impact in the prevention of cytokine storm, which is the principal cause of death associated with severe COVID-19 pneumonia (173). In perspective, estrogen levels appear capable of modulating lung inflammation and damage, and potentially affect the outcomes of respiratory diseases such as SARS-CoV-2 pneumonia (174). Estrogens, and to a lesser extent also progesterone, modulate the release of cytokines, as well as proliferation, differentiation, and polarization in diverse immune cell lineages. The use of antiestrogens (tamoxyfen, toremifene) may interfere with differentiation and maturation of dendritic cells (175).

Some experts see MHT as a plausible part of therapeutic strategy aimed at restoring immunological tolerance and curbing the cytokine storm during coronavirus infection (176).

## MHT

For the adequate clinical management of patients during the period of menopausal transition and early postmenopause, it is necessary to adhere to general criteria for the stages of female reproductive system aging STRAW (Stages of Reproductive Aging Workshop), developed in 2001 and revised in 2011, incorporating the results of large cohort studies conducted over the first decade of the new millenium (STRAW+10). The criteria were developed on the basis of studying the relationship between changes in hormonal parameters and the characteristics of menstrual cycle, which is extremely important for clinical practice when choosing therapy. Despite the universality of endocrine changes during reproductive aging, different stages of this process may differ individually in duration and be accompanied by various specific symptoms (vasomotor, psycho-emotional, vaginal, sexual, etc.) and systemic disorders like the loss of bone mass, unfavorable cardiovascular risk profile associated with the development of visceral obesity, dyslipidemia, endothelial dysfunction, impaired glucose tolerance, etc. (177–180).

Currently, all leading international menopause societies recommend starting MHT in the peri- and postmenopausal period, at an age younger than 60 years and at menopause duration less than 10 years, when the benefit/risk ratio of MHT is most favorable in terms of relieving menopausal symptoms and preventing osteoporosis (181–185). The purpose of using MHT in peri- and postmenopausal women is to partially compensate for the sex hormone deficit. MHT is the most effective treatment for vasomotor symptoms and prevention of postmenopausal osteoporosis in women under the age of 60 years. A wide variety of MHT formulations are currently available worldwide, differing in components, doses, and forms (**Table 3**). The main components of MHT are estrogens and progestogens. Women after hysterectomy are prescribed MHT containing estrogens only. Micronized progesterone or synthetic progestogens are added to MHT for women with intact uterus to reduce the risk of endometrial hyperplasia and carcinoma conferred by estrogen monotherapy (188).

**Table 3:** Commonly prescribed hormone therapies.

Preparation	Doses	Comments
**Systemic estrogen therapies** ^a^ **Oral estrogen tablets** Micronized E2 Estradiol valerate^b^ Conjugated Equine Estrogens	0.5, 1.0, 2.0 mg/d 1.5 mg/d 0.3, 0.45, 0.625 mg/d	Higher doses available Preparation used in WHI
**Transdermal estrogens** Estradiol patch	0.025 to 0.1 mg once or twice weekly depending on preparation 0.014 mg/wk	Corresponds to 0.5 to 2.0 mg estradiol tablets Diffusion can be different from one patch to another Preserved bone in women >60 y old
Estradiol percutaneous gel	0.25–1.5 mg qd	Corresponds to 0.5 to 2.0 mg estradiol tablets Can be transferred to persons and pets by skin contact
Estradiol transdermal spray	1.5 mg qd	Estradiol *via* spray Can be transferred to persons and pets by skin Contact
Vaginal ring Estradiol acetate	0.05–0.10 mg/d	Systemic levels of estradiol provide relief of vasomotor symptoms; 90-d duration/ring
**Progestogen therapies** **Oral progestin tablets** Medroxyprogesterone acetate Norethindrone Neta Megestrol acetate Dydrogesterone^b^ Chlormadinone acetate^b^ Medrogestone^b^ Nomegestrol acetate^b^ Promegestone^b^	2.5, 5, 10 mg/d 0.35 mg/d 5.0 mg/d 20, 40 mg/d 10 mg/d 5, 10 mg/d 5 mg/d 3.75, 5 mg/d 0.125, 0.25, 0.5 mg/d	Utilized in WHI
**Oral progesterone capsule** Micronized progesterone	100, 200 mg/d	In peanut oil; avoid if peanut allergy. May cause drowsiness and should be taken at bedtime
Intrauterine system progestin^c^ LNorg	20 μg released/d 6 μg/d	IUD for 5-y use IUD for 3-y use
Vaginal gel progesterone^c^	4%, 8%	45- or 90-mg applicator
**Combination hormone therapies** **Oral** CEE + MPA E2 + progesterone E2 + Neta E2 + drospirenone E2 + norgestimate E2 + dydrogesterone^b^ E2 + cyproterone acetate^b^ E2 + MPA^b^ CEE + BZA^d^	0.3–0.625 mg/1.5–5 mg/d 1 mg/d 0.5–1 mg/0.1–0.5 mg/d 0.5–1 mg/0.25–1 mg/d 1 mg/0.09 mg/d 1–2 mg/5–10 mg/d 2 mg/1 mg/d 1–2 mg/2–10 mg/d 0.45 mg/20 mg/d	Cyclic or continuous Continuous Continuous Continuous Cycle 3 d E alone, 3 d E+ progesterone Cyclic and continuous Continuous Continuous Continuous
**Transdermal** E2 + Neta E2 + LNorg	50 μg/0.14–0.25 mg/patch 45 μg/0.015 mg/patch	Twice weekly Once weekly
**Synthetic steroid** Tibolone	2,5 mg/d	Continuous Not earlier than 12 months after the last menstruation or immediately after bilateral ovariotomy

IUD, intrauterine device; E, estrogen; E2, 17-b estradiol; LNorg, levonorgestrel; Neta, norethindrone acetate or norethisterone acetate; qd, once daily.

a Not all preparations and doses are available in all countries.

b Only available outside the United States.

c Not approved in the United States for endometrial protection when administered with postmenopausal estrogen.

d Approved indications in the United States include treatment of moderate to severe vasomotor symptoms associated with menopause and prevention of postmenopausal osteoporosis. In the European Union, the indications state: treatment of estrogen deficiency symptoms in postmenopausal women with a uterus (with at least 12 mo since the last menses) for whom treatment with progestin-containing therapy is not appropriate. The experience treating women older than 65 years is limited (182, 186, 187).

The combined estrogen-gestagenic therapy in a cyclic regime is prescribed to women with intact uterus in perimenopause, but not earlier than 6 months after the last menstruation, as a treatment for menopausal symptoms and prophylaxis of postmenopausal osteoporosis (181, 183, 185, 189). The monophasic combined low-dose and ultra-low-dose continuous estrogen-progestogen therapy is recommended for postmenopausal women with intact uterus (12 months after the last menstrual period) (181, 183, 185, 190). Estrogen delivery methods can be oral, transdermal (patches, gels, and sprays), subcutaneous (implants), and vaginal, whereas progestogens can be delivered orally, transdermally, or intrauterally. Tibolone is a synthetic steroid with estrogenic, progestogenic, and weak androgenic activities, indicated for the treatment of menopausal syndrome in postmenopausal women (186). An additional advantage of this drug is the absence of proliferative activity in relation to the endometrium and mammary glands, as well as a significant effect on the growth of myoma nodules. Tibolone has been associated with negligibly increased risks of breast cancer (191, 192) and decreased risks of venous thromboembolism (VTE) (181, 184, 186, 193) (**Table 4**).

**Table 4:** Potential risks of MHT.

Breast cancer	Increased risks of breast cancer have been associated with MHT used for longer than 5 years and involving certain formulations (conjugated equine estrogens plus medroxyprogesterone acetate). The actual risk of breast cancer among MHT users is estimated to be less than 0.1% per year or less than 1 case per 1000 woman-years. MHT with micronized progesterone or dydrogesterone has been associated with a lower risk of breast cancer compared to other progestogens (184, 192, 194, 195). Local administration of the therapy is breast cancer risk-neutral (196–199).
VTE	The risk of VTE is significant in women having started MHT before the age of 60 within 10 years of menopause (200). The absolute risk of VTE in women under 60 years of age is generally low (184). In women at increased risk of VTE, transdermal estrogens are a safer choice than oral agents, especially when combined with micronized progesterone (198, 199, 201).
Ischemic stroke	No extra risk burden for low-dose transdermal estrogen and a dose-dependent increase in risk burden for oral estrogen recipients in high-risk cohorts (199, 202, 203).
Endometrial cancer	Increased risk in patients with intact uterus on estrogen monotherapy, low risks for cyclic combination MHT, and no extra risks posed by continuous combination regimens (196).
Ovarian cancer	Evidence from randomized controlled trials suggests no increased risk of ovarian cancer associated with menopausal hormone therapy (196).

The choice of MHT should be personalized by accounting for risk factors, including cardiovascular diseases, VTE, breast cancer, and postmenopausal osteoporosis, and comorbidities. The main principle is selection of minimum effective dosage, determination of optimal dosage and form of MHT, and the choice of a regimen accounting for physical age, the stage of reproductive aging (STRAW+10), and the needs of the patient (184). In perimenopause, standard (2 mg) and low doses (1 mg) of estradiol as part of MHT are used, in postmenopause, low and ultra-low doses (0.5 mg) of estrogens are used. In postmenopausal women, the reference level of estradiol is ≤ 10 pg/mL and varies with age, presence of vasomotor symptoms and vulvovaginal atrophy, and body mass index (204). In a study evaluating serum estradiol levels in postmenopausal women using MHT, when using estradiol hemihydrate or estradiol valerate serum estradiol levels increased with increasing dose of the drug, however, the degree of increase was not directly proportional to the dose; in particular, for oral estradiol, increasing the dose from 1 to 2 mg resulted in an increase of serum estradiol levels to approximately 60% instead of a doubling. This finding suggests that “low doses” of estrogen may be adequate from the start of MHT (205). Data from the Women’s Health Initiative (WHI) randomized controlled trial and other studies support the safe use of MHT for at least 5 years in healthy women commencing treatment under the age of 60 while being less than 10 years in postmenopause. The question of continuing therapy is decided individually, taking into account the possible risks (184). The North American Menopause Society experts published a statement in 2015 on the possible continuation of the use of MHT at the lowest effective dose in women over 65 years of age for the treatment of persistent hot flashes, given that the patient has received detailed information about the possible risks and is under close medical supervision (206).

### MHT reshapes immunological parameters

The gradual decline of ovarian function with age is a physiological process, but only in a small percentage of women the perimenopause proceeds unnoticed. Estimated 50–82% of women develop a symptom complex called “climacteric syndrome” with early vasomotor manifestations. The broader concept of climacteric syndrome involves psychoemotional and somatic components, as well as the increasing risk of cardiometabolic and cognitive dysfunctions in the long-range. Pathogenetic treatment of the climacteric syndrome aims to compensate the deficiency of sex hormones, first and foremost estrogens; the strategy allows slowing down the progression of the deficiency and thereby delay the onset of organic changes in target tissues and systems of the female body. Partial replenishment of the sex hormone deficiency slows down natural aging and eliminates long-term consequences.

The choice of MHT regimen depends on the stage of reproductive aging; the possibilities include combined estrogen-gestagen therapy in a cyclic mode using biphasic drugs in perimenopausal patients with intact uterus; monophasic combination therapy in postmenopausal patients with intact uterus, and estrogen monotherapy in hysterectomized patients. The modern principles of MHT prescription provide for an assessment of the risks of VTE individually for each patient. In postmenopausal women with low risk of VTE, estrogen in MHT could be administered both orally and transdermally at the lowest effective doses (low/ultra-low), according to existing recommendations (2016 IMS Recommendations on women’s midlife health and menopause hormone therapy) (184). For patients with increased risk of VTE and indications for MHT, transdermal forms of estrogens are prescribed as part of MHT, while in women with a history of VTE, MHT is contraindicated. During the pandemic in Russia, patients on MHT and infected with SARS-Cov-2 are prescribed anticoagulants to prevent VTE according to the guidelines ``Prevention, diagnosis and treatment of a new coronavirus infection (COVID-19)” 2021 and local clinical protocol for the Treatment of Patients with a New Coronavirus Infection (COVID-19). Summing up the available data, our personal opinion as the authors of this review is that MHT should not be cancelled in case of COVID-19 infection.

A complex relationship between sex hormones and immune system implies that MHT can exert pleiotropic effects on immunity in peri- and postmenopausal patients (207). The majority of studies focusing on such effects demonstrate a decrease in the production of pro-inflammatory cytokines (TNF-α, IL-1β, IL-6) by peripheral blood mononuclear cells of MHT recipients *ex vivo* or *in vivo* (208).

The influence of MHT on systemic inflammatory status may partially depend on estrogen administration routes. For instance, transdermal estrogens attenuated the response of the hypothalamic-pituitary-adrenal axis to low doses of endotoxin *in vivo*. The effect was accompanied by alleviation of the endotoxin-induced expression of pro-inflammatory cytokines IL-6 and TNF-α, as well as IL-1 receptor antagonist (IL-1ra). Oral administration of the same doses failed to reproduce this effect, probably due to the primary passage of the ingested hormones through the liver, where they triggered production of C-reactive protein and other pro-inflammatory molecules (209–211).

Clinical research data suggest that surgical menopause (upon ovariectomy) is accompanied by immunodeficiency with a decline in B cell counts and decreased serum levels of interferon γ. More generally, the rates of cellular and humoral immune response in women on MHT are higher than in matching controls (212). The definite immunological benefits of perimenopausal MHT confirm the important role of estrogens in regulation of immune functionalities (207). At the same time, it should be admitted that our understanding of MHT effects on immunological parameters is still fragmentary.

### MHT and COVID-19

In 2020, at the first peak of the pandemic, the Italian Society of Contraception [Società Medica Italiana per la Contraccezione] issued the following opinion: MHT, as well as combined oral contraception regimens, should not be discontinued in patients with mild and moderate symptomatic COVID-19, whereas in severe cases the use of these medications is superfluous and should be replaced with anticoagulant therapy upon aggravation (213). Concomitantly, a board of experts representing specialized medical societies of Spain [Asociación Española para el Estudio de la Menopausia; Sociedad Española de Ginecología y Obstetricia; Sociedad Española de Trombosis y Hemostasia] recommended switching to parenteral routes of MHT administration, continue with parenteral MHT while adding anticoagulants, and fully replace MHT with anticoagulant therapy in, respectively, mild, moderate, and severe symptomatic COVID-19 (214). Large-scale self-monitoring data from the UK-based COVID-19 symptom tracking app showed lower incidence of hospitalization and reduced need for respiratory support in MHT recipients compared with matched controls (7, 215). A recent population study enrolling 151,193 MHT recipients and 152,637 matching non-recipients recorded significantly lower risks of adverse COVID-19 outcome for the former (adjusted odds ratio 0.22, 95% confidence interval 0.05–0.94) (7);. In a retrospective study, MHT reduced the risks of lethal COVID-19 outcomes in women over 50 by over 50% (odds ratio 0.33, 95% confidence interval 0.18–0.62; hazard ratio 0.29, 95% confidence interval 0.11–0.76) (8). Another retrospective study conducted in Sweden confirmed the reduced lethality of COVID-19 among postmenopausal MHT recipients (216). Given the design of the study, randomization was not considered. The weakness of the study is the lack of indication of the duration of endocrine/MHT intake. The level of sex hormones in postmenopausal patients was not determined, however, the distribution into groups suggested that in patients receiving endocrine therapy for breast cancer, the level of estrogen was reduced; in patients receiving MHT, estrogen levels were elevated; and in a control group of postmenopausal patients, without treatment, estrogen levels were consistent with postmenopausal values. A phase 2 clinical trial for possible alleviation of COVID-19 symptom severity through administration of transdermal estrogen patches was launched in the USA (NCT04359329).

Some experts argue that caution should be exercised regarding claims that the skewed sex ratio of COVID-19 morbidity and associated mortality is really determined by circulating steroid hormones. For instance, congenital genetic breakdowns in the immune system are arguably more impactful with regard to the critically aggravated course of the disease. The purposeful use of estrogens and progesterone in COVID-19 remains an intuitive concept that has not been supported by biochemical, physiological, or clinical evidence (217). Some authors believe that genetics and innate immune system disorders are more relevant to the sex-based difference in COVID-19 mortality than the circulating steroid hormone levels. Noteworthy, heritable defects in IFN-I immunity responsible for the deployment of life-threatening pneumonia in COVID-19 were detected in at least 2.6% of women and 12.5% of men (217). Bastard et al. hypothesized that congenital defects of cytokine system may contribute to the observed difference in disease severity between women and men. Apparently, germline mutations in IFN-I genes and neutralizing autoantibodies against corresponding proteins may underlie the diversity of respiratory complications (218). As reported by Zhang et al., at least 3.5% of patients with life-threatening COVID-19 pneumonia carry either autosomal recessive mutations in *IRF7* or *IFNAR1*, autosomal dominant mutations in TLR3, TICAM1, TBK1 or IRF3, or *de novo* autosomal dominant mutations in UNC93B1, IRF7, IFNAR1 or IFNAR2 (219).

The plausible effects of MHT on the incidence and lethality of COVID-19 need further investigation. Although sex steroids appear to play an important role in modulating susceptibility to SARS-CoV-2, they cannot fully account for the skewed demographic patterns of COVID-19 mortality, indicating that other causes and mechanisms are yet to be understood (220).

## Conclusion

**Figure 1:**
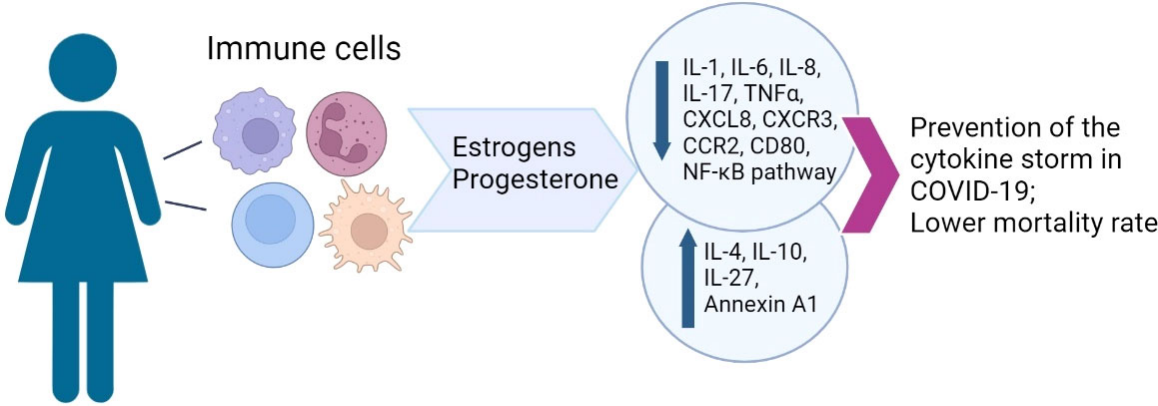
A graphical summary of sex hormone effects on female immunity.

High physiological concentrations of estrogens and progesterone synergistically reduce the production of pro-inflammatory cytokines by innate immune cells and also promote the anti-inflammatory response of T cells and immune tolerance, while stimulating the antibody production by B cells. In COVID-19, MHT may mitigate the clinical symptoms while increasing the antibody production (92). This knowledge is clinically relevant, as MHT is quite common, while dedicated development of new antivirals is hampered by the ongoing pandemic. Specific effects of MHT on hemostasis require careful assessment for the risks of its continued use in symptomatic COVID-19. The orchestrating role of estrogens in immune response and their protective effect on vascular endothelium should not be neglected. The correlation of sex steroid levels with severity and lethality of the disease indicates the potential of using hormone therapy to modulate the immune response and increase the resilience to adverse outcomes (9) (**Figure 1**). The overall success of MHT is based on decades of experience in clinical trials. According to the current standards, MHT should not be discontinued in COVID-19 with the exception of critical illness.

## Author contributions

Writing—original draft preparation, МA, PV, and SY; writing—review and editing, МA, PV, SY, OY, TF, AE, and GS. All authors have read and agreed to the published version of the manuscript.

## Funding

The research was performed within the framework of State Assignment No 121032500100-3. Part of the work concerning monocytes was supported by a grant for young Russian scientists MK-1573.2022.3. This work was supported by Russian Science Foundation [grant number 22-15-00241].

## Acknowledgments

We acknowledge Natalia Usman for helpful discussions.

## Conflict of interest

The authors declare that the research was conducted in the absence of any commercial or financial relationships that could be construed as a potential conflict of interest.

## Publisher’s note

All claims expressed in this article are solely those of the authors and do not necessarily represent those of their affiliated organizations, or those of the publisher, the editors and the reviewers. Any product that may be evaluated in this article, or claim that may be made by its manufacturer, is not guaranteed or endorsed by the publisher.
